# Fruit and Vegetable Consumption and Frailty: A Systematic Review

**DOI:** 10.1007/s12603-018-1069-6

**Published:** 2018-06-26

**Authors:** Gotaro Kojima, C. Avgerinou, S. Iliffe, S. Jivraj, K. Sekiguchi, K. Walters

**Affiliations:** 1 Department of Primary Care and Population Health, University College London (Royal Free Campus), Rowland Hill Street, London, NW3 2PF UK; 2 Department of Epidemiology and Public Health, University College London, London, UK; 3 Department of General Medicine, Shinshu University School of Medicine, Matsumoto, Nagano, Japan

**Keywords:** Frailty, fruits, vegetables, nutrition, diet

## Abstract

**Objective:**

To identify currently available evidence on fruit and vegetable consumption in association with frailty by conducting a systematic review of the literature and to summarise and critically evaluate it.

**Design:**

Systematic review.

**Setting:**

Four electronic databases (Embase, MEDLINE, CINAHL and PsycINFO) were systematically searched in August 2017 for observational cohort studies providing cross-sectional or prospective associations between fruit and vegetable consumption and frailty risks. Additional studies were searched by manually reviewing the reference lists of the included studies and related review papers and conducting forward citation tracking of the included studies. The methodological quality of prospective studies was assessed using the Newcastle-Ottawa scale.

**Participants:**

Community-dwelling general populations.

**Results:**

A total of 6251 studies were identified, of which five prospective studies with follow-up periods of 2–10.5 years and two cross-sectional studies were included. Among the five prospective studies, three had adequate methodological quality. Because of different measurements and statistical methodologies, a meta-analysis was not possible. The two studies of good quality showed that fruit and vegetable consumption was mostly associated with lower risk of incident frailty. The other study as a sub-analysis retrospectively examined baseline fruit and vegetable consumption of those who developed frailty and those who did not at follow-up and showed no significant associations.

**Conclusions:**

Although good quality studies on this topic are scarce, there is some suggestion that higher fruit and vegetable consumption may be associated with lower frailty risk. More high quality research is needed.

**Electronic Supplementary Material:**

Supplementary material is available for this article at 10.1007/s12603-018-1069-6 and is accessible for authorized users.

## Introduction

Frailty, a geriatric syndrome characterised by an agerelated decrease in physiological reserve and an increase in vulnerability to stressors, commonly affects older people (1). Approximately 10% of persons aged 65 years or older and at least a quarter of those aged over 85 years are frail (2). Frailty is associated with various negative health outcomes, including falls, fractures, hospitalization, nursing home placement, disability, dementia, impaired quality of life and mortality (1). Due to the ageing world population, the number of frail older people is projected to increase (1). In light of the serious consequences of frailty, it is a priority of all healthcare professionals to prevent the development of frailty and delay its progression. For these purposes, an effective strategy is required to identify significant risk factors for frailty, which would lead to effective interventions or treatments.

In recent years, different aspects of diet have been studied in frailty research (3). Intakes of various macro- and micronutrients as well as healthy dietary patterns, such as Mediterranean diet, have been found to be associated with lower frailty risks (4, 5). However it is not well-established what components within these broad dietary patterns contribute to this association. Fruits and vegetables are recognised as an important part of a healthy diet for all ages. Fruits and vegetables are important sources of vitamins, mineral, fibre, anti-oxidants and anti-inflammatory agents, and guidelines recommend adequate amount should be consumed (6). Increased fruit and vegetable intakes are associated with a lower risk of cardiovascular diseases (7, 8), various types of cancer (9, 10), and mortality (11). Although it can be hypothesised that fruit and vegetable intake is also beneficial against frailty, the body of knowledge on the association between fruit and vegetable intake and frailty in the literature is conflicting and not well synthesised (3). Therefore, we aimed to identify currently available evidence on fruit and vegetable consumption in association with frailty by conducting a systematic review of the literature and to summarise and critically evaluate it.

## Method

### Data source and search strategy

A systematic review of the literature was performed in August 2017 based on a protocol (PROSPERO registration number: CRD42017057165) developed a priori according to the Preferred Reporting Items for Systematic Review and Meta- Analyses (PRISMA) statement (12). Four electronic databases (Embase, MEDLINE, CINAHL Plus and PsycINFO were systematically searched with explosion functions if available between 2000 and August 2017. The beginning of the search period, 2000, was chosen because the Cardiovascular Health Study frailty criteria, the most widely used frailty criteria, were published in 2001 (13). No language restriction was imposed. We used a combination of Medical Subject Heading (MeSH) terms and text keywords as follows: Fruit (MeSH) OR Vegetables (MeSH) OR Fruit Vegetable(s) (MeSH) OR Fruit and Vegetable Juice(s) (MeSH) Fruit Juice(s) (MeSH) OR Vegetable Juice (MeSH) OR Antioxidant(s) (MeSH) OR Diet(s) (MeSH) OR Diet Therapy (MeSH) OR Nutrition (MeSH) OR Nutrition Therapy (MeSH) OR fruit* OR vegetable* OR anti-oxidant* OR antioxidant* OR diet* OR nutrition* AND frailty related terms, including Frail Elderly (MeSH) OR Frailty Syndrome (MeSH) OR frail*. The reference lists of the included studies and related review papers were manually searched for additional studies. The forward citation tracking of the included studies was conducted using Google Scholar (http://scholar.google.com/).

### Study selection

Any original papers of observational cohorts providing cross-sectional or prospective associations between fruit and vegetable consumption and frailty were considered. Selective samples unrepresentative of community-dwelling people in general, such as hospitalised patients or those with heart failure, were excluded. Studies reporting fruit and vegetable consumption as a quantity or the consumption frequency of fruits alone, vegetables alone or fruits and vegetables combined were included. Those including a specific type of fruit or vegetable only, or those concerned with dietary patterns including fruit and vegetable consumption as part of a wider diet including other nutrients (e.g. the Mediterranean diet) were excluded unless they reported on the associations between fruit and/or vegetable consumption and frailty separately. To be included, studies had to define frailty by original or modified version of validated criteria designed to measure frailty. Randomised controlled trials, reviews, conference abstracts, editorials, comments and letters were excluded. One author (GK) first screened for eligibility all study titles and then the abstracts and full texts of the studies identified by the systematic review. The second author (CA) independently screened the full-texts for eligibility. We solved any disagreement by discussion.

### Data extraction

A standardised data collection form was used to extract data including first author, publication year, cohort name, location, sample size, proportion of women, age, frailty criteria, follow-up period, fruit and vegetable measurement method and findings.

### Methodological quality assessment

The methodological quality of prospective studies was assessed by two authors (GK and KS) independently using the Newcastle-Ottawa scale for cohort studies (14), which consists of nine items covering three domains: Selection (representativeness of the exposed cohort; selection of the nonexposed cohort; ascertainment of exposure; and demonstration that outcome of interest was not present at start of study), Comparability (comparability of cohorts on the basis of the design or analysis) and Outcome (assessment of outcome; was follow-up long enough for outcomes to occur; and adequacy of follow-up of cohorts). A study meeting five items or more was considered to have adequate methodological quality. Disagreements were solved by discussion.

### Data analysis

We aimed to conduct a meta-analysis to combine findings of the included studies if it was possible, otherwise, however, we would pursue a narrative review.

## Results

### Selection process

Supplementary Figure is the PRISMA flowchart showing the study selection process and results of the systematic review. The search of the four databases identified a total of 6251 studies. After excluding duplicates and studies that were considered not eligible through screening of the titles and abstracts, full-texts of nine potentially eligible studies were reviewed. Two studies were excluded because these studies did not examine fruit and vegetable consumption but dietary patterns, leaving seven studies for this review.

### Study characteristics

Table 1 shows the characteristics of the seven included studies (15-21). Five studies were prospective with follow-up periods of 2–10.5 years (15-19) and two studies were crosssectional (20, 21). One study each was from France (16), Spain (15) the US (18), the UK (19), Netherlands (20) and Japan. (21) One study used a combination of three cohorts (Three-City Study, the Senior-ENRICA and the Integrated Multidisciplinary Approach cohorts) (17). The Three-City Study and the Senior- ENRICA cohorts were also used individually by Rahi et al. and Leon-Munoz et al., respectively (15, 16). The sample sizes ranged from 432 (18) to 2926 (17). The proportion of female participants ranged from 27.9% (19) to 100% (21). All studies used middle-aged and elderly populations; the mean age varied considerably from 50’s to 80’s. The modified versions of the Cardiovascular Health Study frailty criteria (13) were used by five studies (15-17, 19, 21) to define frailty while one study (18) used FRAIL scale and another study (20) used Tilburg Frailty Indicator. The data collection methods of fruit and vegetable consumption were based on questionnaires (16, 18-21), either self-reported or by a research personnel (15, 17). Different measurements of fruit and vegetable consumption were employed: the number of portions per day (17), the number of times per day (16, 1), quantity in grams per day (15, 21) and whether consuming daily or not (YES/NO) (19, 20). Due to the various measurements of fruit and vegetable consumption and the definitions of frailty as well as differing statistical methodologies (logistic regression, linear regression, t-test), a meta-analysis was not possible, and a narrative synthesis was performed.

**Table 1:**
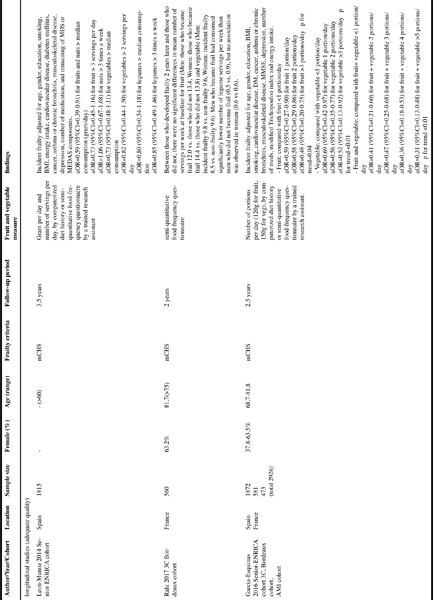

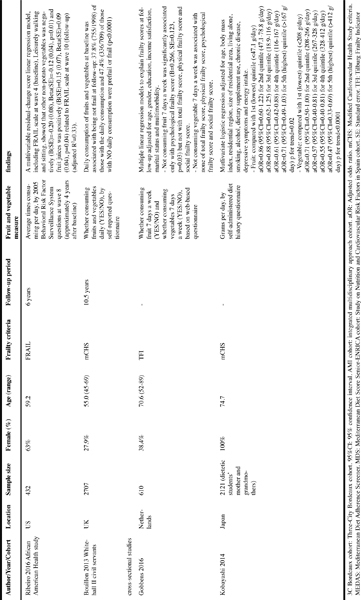
Summary of studies examining associations between fruit and vegetable consumption and frailty

### Methodological quality assessment of prospective studies

Three studies of incident frailty were both considered to have adequate methodological quality (15-17). The remaining two studies were considered to have suboptimal quality (18, 19). (Supplementary Table)

### Prospective studies (adequate methodological quality)

#### Leon-Munoz et al. (15)

Among 1,815 Spanish older people from the Seniors- ENRICA study, consuming the median amount or more of fruits and nuts was associated with lower risk of incident frailty over a 3.5-year period Odds ratio (OR)=0.59, 95% confidence interval (CI)=0.39-0.91) compared with comsuming less than the median. However consuming three servings or more of fruit per day was not (OR=0.73, 95%CI=0.45-1.16). Neither consuming two servings or more of vegetables per day (OR=0.82, 95%CI=0.44-1.50) nor consuming the median amount or more of vegetables (OR=0.73, 95%CI=0.48-1.11) were associated significantly with frailty risk. The median amounts of fruit and nuts and vegetables were not provided in this paper. All models were adjusted for age, gender, education, smoking, body mass index, energy intake, cardiovascular disease, diabetes, cancer, asthma or chronic bronchitis, musculoskeletal disease, depression, number of medications and the other components of the Mediterranean Diet Score or Mediterranean Diet Adherence Screener Score.

#### Rahi et al. (16)

This study followed 560 non-frail French older people from the Three-City study and found that higher Mediterranean diet adherence based on Mediterranean Diet Score at baseline was associated with lower incident frailty risk over 2-year followup. As a sub-analysis, baseline values of nine components of the Mediterranean Diet Score, namely mean numbers of weekly servings of (1) legumes, (2) cereals, (3) seafood, (4) meat, (5) dairy products, (6) fruits (7) and vegetables, “frequent” or “all the time” use of (8) olive oil and “mild-to-moderate” consumption of (9) alcohol, were retrospectively examined according to follow-up frailty status (frail vs. non-frail) using t-test or chi-square tests. There were no statistical differences in mean numbers of weekly servings for fruit (Men: those who developed frailty 12.0 vs. those who did not 13.4, Women: those who developed frailty 14.4 vs. those who did not 13.8) and vegetable (Men: those who developed frailty 9.8 vs. those who did not 9.6, Women: those who developed frailty 8.5 vs. those who did not 9.6). Legumes were significantly more frequently consumed by non-frail men than frail men while no such associations were observed in women. (Men: those who developed frailty 0.5 vs. those who did not 0.9, Women: those who developed frailty 0.6 vs. those who did not 0.6). It should be noted that statistical power may have been lowered by dividing the cohort into smaller groups: 19 men and 60 women who developed frailty and 187 men and 294 women who did not.

#### Garcia-Esquinas et al. (17)

Incident frailty risks according to fruit and vegetable consumption at baseline were investigated in a total of 2,926 older men and women who were free of frailty at baseline from three different cohorts (Three-City Bordeaux cohort and the Integrated Multidisciplinary Approach cohort from France and Seniors-ENRICA cohort from Spain). The modified Cardiovascular Health Study frailty criteria were used to define frailty. Those who consumed higher amounts of fruit, vegetables and both combined had a significantly lower risk of developing frailty over 2.5 years. The effects were dose-dependent and ORs of incident frailty controlled for age, gender, education, body mass index, smoking, cardiovascular disease, diabetes, cancer, asthma or chronic bronchitis, musculoskeletal disease, cognition, depression, number of medications, modified Mediterranean Diet Score and energy intake were: for those who consumed 1, 2 or >3 portions of fruit/day (1 portion=120g of fruits), compared with those who consumed <1 portion/day, 0.59 (95%CI=0.27-0.90), 0.58 (95%CI=0.29-0.86) and 0.48 (95%CI=0.20-0.75), respectively (p for trend=0.04); for those who consumed 1, 2 or >3 portions of vegetables/day (1 portion=150g of vegetables), compared with those who consumed <1 portion/day, 0.69 (95%CI=0.42- 0.97), 0.56 (95%CI=0.35-0.77) and 0.52 (95%CI=0.13-0.92), respectively (p for trend<0.01); and for those consumed 2, 3, 4 and >=5 portions of fruits and vegetables combined/ day, compared with those who consumed <=1 portion, 0.41 (95%CI=0.21-0.60), 0.47 (95%CI=0.25-0.68), 0.36 (95%CI=0.18-0.53) and 0.31 (95%CI=0.13-0.48), respectively (p for trend<0.01).

### Prospective studies (suboptimal methodological quality)

#### Ribeiro et al. (18)

A US study by Ribeiro et al. examined baseline fruit and vegetable consumption and changes in frailty status measured by the FRAIL scale over a 6-year period between 2004 and 2010 in 432 middle-aged and older African American men and women. Frequencies of five types of fruit and vegetable intakes (average times taken per day) were measured in 2006 based on a questionnaire: (1) fruit juices such as orange, grapefruit or tomato, (2) fruit, (3) green salad, (4) carrots and (5) vegetable different from carrots, potatoes or salad (defined as “other non-potato vegetables”). All these variables, physical activity levels, age, gender and baseline FRAIL scale were initially entered into a multivariable residual-change score linear regression model to predict FRAIL scale at follow-up. After backward stepwise elimination of non-significant variables, the final model included other non-potato vegetables, fruit juices, leisurely walking, sitting and baseline FRAIL scale (adjusted R^2^=0.33). The intake of other non-potato vegetables was negatively (B(SE)=-0.20 (0.08), Beta(SE)=-0.12 (0.04), p=0.01) but consumption of fruit juices was positively (B(SE)=0.15 (0.07), Beta(SE)=0.09 (0.04), p=0.04) associated with FRAIL scale. Important confounding factors, such as education or socioeconomic factors, were not considered in the model.

#### Bouillon et al. (19)

Bouillon et al. used the Whitehall II study cohort consisting of 2,707 middle-aged and older civil servants aged 45–69 in the UK to examine the frailty risk over a long follow-up period of 10.5 years. Those who answered that they consumed fruits and vegetables daily in a self-reported questionnaire at baseline were less likely to be frail (37.8%, 755/1998) than those who reported not consuming fruits and vegetables daily (47.4%, 336/709) (p<0.0001). There are some important limitations to be noted. First, the cohort used was a selected sample of civil servants. Second, frailty was measured at follow-up but not at baseline. Baseline frailty status should have been considered in the analysis, or frail participants at baseline should have been excluded if incident frailty had been examined, otherwise reverse causality cannot be denied. Lastly, the presence or absence of daily fruit and vegetable consumption is a rather crude predictor variable.

### Cross-sectional studies

#### Gobbens et al. (20)

A cross-sectional study of 610 middle-aged and older men and women aged 52–89 years (mean age 70.6) in the Netherlands examined associations of fruit and vegetable consumption with frailty, measured by the Tilburg Frailty Indicator. The information was collected via a web-based questionnaire. Multiple linear regression models adjusted for age, gender, education, income satisfaction, marital status and multimorbidity showed that consuming fruits on fewer than 7 days a week was significantly associated only with higher psychological frailty score (B=0.266, SE=0.123, p=0.03) but not with the total, physical and social frailty scores, compared with consuming fruits on 7 days per week. Consuming vegetables on fewer than 7 days per week was associated with none of the frailty scores, compared with consuming vegetables on 7 days per week. As the authors acknowledged, the major limitations included restriction of the sample to those who had internet access and were able to complete the online questionnaire and the crude measurement of fruit and vegetable consumption as a dichotomous variable instead of quantitatively or in a dose-response manner.

#### Kobayashi et al. (21)

Another cross-sectional study used a selected cohort of 2,121 Japanese older women with mean age of 74.7 years old, who were mothers or grandmothers of dietetic students, to examine associations between consumption of fruits and vegetables and frailty. The consumption of fruits and vegetables was measured using a self-administered diet history questionnaire, and frailty was defined by the Cardiovascular Health Study criteria (13)with Woods’ modification (22). Multivariable logistic regression models controlled for age, body mass index, residential region, size of residential area, living alone, smoking, alcohol, dietary supplement use, chronic disease, depressive symptoms and energy intake showed higher intakes of fruits (compared with 1st quintile (lowest), adjusted OR=0.86, 0.88, 0.61 and 0.71 for 2nd-5th (highest) quintiles, respectively) and vegetables (compared with 1st quintile (lowest), adjusted OR=0.71, 0.57, 0.55 and 0.47 for 2nd-5th (highest) quintiles, respectively) were associated with lower frailty risks in a graded manner (p for trend=0.02 and <0.0001, respectively).

## Discussion

This systematic review has identified a total of seven studies examining middle-aged and older populations for associations between fruit and vegetable consumption and frailty. Among three studies with adequate methodological quality, only one study primarily examined fruits and vegetables and showed that higher intakes of fruits, vegetables and both combined were significantly associated with lower incident frailty risks in a dose-response manner (17). The main focus of the other two studies was a Mediterranean diet (15, 16) and fruits and vegetables were examined only in sub-analyses, which showed only fruits and nuts of more than median amount was associated with lower incident frailty risk in one study (15). p ]The findings of two prospective studies with suboptimal quality were consistent: a higher non-potato vegetable intake was associated with lower frailty risks (18), and those who consumed fruits and vegetables daily had lower frailty risks compared with those who did not (19). The former study (18) also showed fruit juice intake at baseline was associated with worse frailty at follow-up. This could be because “fruit juice” described in this study was not restricted to 100% pure fruit juice but could refer to drinks with a lower fruit content or with added sugar. In addition, this “fruit juice” may be replacing real fruit intake and therefore underestimate the true fruit consumption.

One cross-sectional study showed significant dose-response reverse association between higher intakes of fruits and vegetables and prevalent frailty (21). Another cross-sectional study showed not consuming fruit 7 days/week was associated with significantly higher psychological frailty score than consuming fruit 7 days/week, while there were no significant associations between not consuming vegetables 7 days/week and frailty scores (20). Due to cross-sectional nature of these two studies, reverse causality may be possible, for example, loss of appetite can be a feature of frailty leading to lower intake of fruit and vegetables.

Although not included in this review, we identified a further study that did not investigate fruit and vegetable consumption specifically but instead examined dietary patterns including fruits and vegetables in association with frailty. A cross-sectional study of 923 elderly Taiwanese aged 65 or older explored a dietary pattern associated with frailty using reduced rank regression analysis and found that fresh fruit had the highest factor loading value (-0.48) and vegetables had the fourth highest one (-0.33), both suggesting strong inverse associations with frailty (23).

Fruits and vegetables are important part of the Mediterranean diet, which is traditionally consumed in the countries surrounding the Mediterranean and is characterised by high intakes of plant-based foods, such as fruits, vegetables, legumes, whole grains and nuts, and low-to-moderate consumption of red meat and wine (24). A few cross-sectional and prospective studies have suggested inverse associations between higher adherence to the Mediterranean diet and lower frailty risks (5, 24). This protective effect of the Mediterranean diet against frailty is not necessarily attributed only to high consumption of fruits and vegetables, but could also be due to the other characteristics of Mediterranean diet, including consumption of more olive oil or canola oil than butter, more nuts and legumes (containing protein) and more spices other than salt, as well as limited intake of red meats, or all of these features combined (24).

Fruits and vegetables are well known to benefit human health and may also protect against frailty. One of the possible mechanisms is through anti-oxidative effects. A recent systematic review has shown that frailty appears to be associated with higher oxidative stress and possibly lower antioxidant- related measurements (25). Fruits and vegetables are natural sources of anti-oxidants, such as vitamin C, vitamin E, carotenoids and selenium (26). These anti-oxidants may reduce or prevent frailty by decreasing reactive oxygen species, which cause damage to DNA, lipids and proteins and induce mitochondrial dysfunction and apoptosis (26). Another explanation is that fruits and vegetables including legumes are potential source of proteins against frailty. Adequate dietary protein intake is essential to increase muscle protein synthesis and improve physical function, and counteract sarcopenia, the age-related loss of muscle mass and strength, a core feature of frailty. Given some fruits and vegetables, such as legumes and nuts, are rich in protein, those with high intakes of fruits and vegetables may obtain more plant-based proteins than those with low intakes of fruits and vegetables (27).

This study has some limitations. The area of diet, especially fruit and vegetable consumption, in relation to frailty is relatively new, and only a limited number of studies were found through the searches. In addition, because the included studies used different measurements of fruit and vegetable consumption and statistical methodologies, a meta-analysis could not be conducted. It was also not possible to know exactly how fruits and vegetables were defined in all studies: some studies separated legumes or nuts from fruits and vegetables (15, 16) while others did not specify the definitions of fruits and vegetables (17-21). Furthermore, it should be noted that not all studies took into account important potential confounders, including socioeconomic status, education and IQ.

The robust methodology employed in accordance with PRISMA statements was the major strength of this review. The systematic literature search was conducted using four electronic databases with a comprehensive and reproducible search strategy using a combination of MeSH and text terms. The identified studies were screened by two independent investigators with a standardised protocol and were assessed for methodological quality.

## Conclusion

The overall evidence regarding the associations between fruit and vegetable consumption and frailty is scarce in the literature and the study settings, statistical methods and findings were heterogeneous. More high quality research is needed in order to elucidate these associations, especially research to confirm the causal relationships. There is some suggestion from limited evidence that higher fruit and vegetable consumption may be associated with a lower risk of frailty. There were no studies showing fruits or vegetables worsen frailty. If intake of fruits and vegetables is beneficial in preventing or reversing frailty, this might be a good target for intervention against frailty given increasing fruits and vegetables consumption is relatively easy and without significant side effects. Future research should also investigate how much of fruits and vegetables is enough to give protection against frailty among older people.

*Funding:* This study was supported by the Sasakawa Foundation Butterfield Awards for UK-Japan collaboration in medicine and health (Application number B111). GK is funded by a University College London (UCL) Overseas Research Scholarship. Neither funder had any influence on the study design, the collection, analysis, and interpretation of data, the writing of the article or the decision to submit it for publication.

*Acknowledgment:* We thank Ms Sophie Pattison, a clinical support librarian at the Royal Free Hospital Medical Library, for supporting the systematic review.

*Conflict of interest:* None.

## Electronic supplementary material

**Supplementary Figure**. PRISMA Flowchart

**Supplementary Table**. Methodological quality assessment using the Newcastle-Ottawa Quality Assessment Scale (cohort studies)

PRISMA 2009 Checklist
