# Supplementary material for: Fruit and Vegetable Consumption and Frailty: A Systematic Review
Source: J Nutr Health Aging. 2018 Jun 26;22(8):1010–7. doi: 10.1007/s12603-018-1069-6 (PMC6182506; doi:10.1007/s12603-018-1069-6)
Supplement: Supplementary file 1 — Supplementary Figure. PRISMA Flowchart [file 12603_2018_1069_MOESM1_ESM.docx]

**Supplementary Figure**. PRISMA Flowchart

0 additional study identified through other sources

6251 studies identified through database searching

Embase (n=3500)

MEDLINE (n=1711)

CINAHL Plus (n=840)

PsycINFO (n=200)

9 articles for full-text review

Total of 6251 studies identified

3833 studies screened for titles and abstracts

3824 studies excluded by title and abstract screening

2418 duplicated studies excluded

5 prospective studies and 2 cross-sectional studies to be included

5 prospective studies for methodological quality assessment

2 studies excluded by full-text review for not using fruit and vegetable consumption but dietary patterns
