# Supplementary material for: Fruit and Vegetable Consumption and Frailty: A Systematic Review
Source: J Nutr Health Aging. 2018 Jun 26;22(8):1010–7. doi: 10.1007/s12603-018-1069-6 (PMC6182506; doi:10.1007/s12603-018-1069-6)
Supplement: Supplementary file 2 — Supplementary Table. Methodological quality assessment using the Newcastle-Ottawa Quality Assessment Scale (cohort studies) [file 12603_2018_1069_MOESM2_ESM.docx]

**Supplementary Table**. Methodological quality assessment using the Newcastle-Ottawa Quality Assessment Scale (cohort studies)

| Author/year | Selection 1 | Selection 2 | Selection 3 | Selection 4 | Comparability 1 | Comparability 2 | Outcome 1 | Outcome 2 | Outcome 3 | total |
| --- | --- | --- | --- | --- | --- | --- | --- | --- | --- | --- |
| Rahi 2017 | 1 | 1 | 1 | 1 | 1 | 1 | 1 | 1 | 1 | 9/9 |
| Garcia-Esquinas 2016 | 1 | 1 | 1 | 1 | 1 | 1 | 1 | 1 | 0 | 8/9 |
| Ribeiro 2016 | 1 | 1 | 0 | n/a | 0 | 0 | 0 | 1 | 0 | 3/8 |
| Leon-Munoz 2014 | 1 | 1 | 0 | 1 | 1 | 1 | 1 | 1 | 1 | 8/9 |
| Bouillon 2013 | 0 | 1 | 0 | 0 | 0 | 0 | 1 | 1 | 1 | 4/9 |
|  |  |  |  |  |  |  |  |  |  |  |
| Cross-sectional |  |  |  |  |  |  |  |  |  |  |
| Gobbens 2016 | - | - | - | - | - | - | - | - | - | - |
| Kobayashi 2014 | - | - | - | - | - | - | - | - | - | - |
